# Functions of Tfh Cells in Common Variable Immunodeficiency

**DOI:** 10.3389/fimmu.2020.00006

**Published:** 2020-01-30

**Authors:** Corentin Le Saos-Patrinos, Séverine Loizon, Patrick Blanco, Jean-François Viallard, Dorothée Duluc

**Affiliations:** ^1^ImmunoConcEpT, CNRS-UMR 5164 and Université de Bordeaux, Bordeaux, France; ^2^Centre Hospitalier Universitaire de Bordeaux, Service d'Immunologie et Immunogénétique, Bordeaux, France; ^3^Centre Hospitalier Universitaire de Bordeaux, Service de Médecine Interne, Hôpital du Haut-Lévêque, Pessac, France

**Keywords:** follicular helper T cells, CVID, complications, B cells, IFNγ

## Abstract

Common variable immunodeficiency is the most common clinical primary immunodeficiency in adults. Its hallmarks are hypogammaglobulinemia and compromised B-cell differentiation into memory or antibody-secreting cells leading to recurrent infections. This disease is heterogeneous, with some patients harboring multiple complications such as lymphoproliferative disorders, autoimmune manifestations, or granulomatous inflammation. The mechanisms leading to these complications remain elusive despite numerous associations found in the literature. For instance, although described as a B cell intrinsic disease, numerous abnormalities have been reported in other immune cell compartments. Here, we tuned our attention to follicular helper T cells, a CD4^+^ T cell population specialized in B cell help, considering the recent publications showing an involvement of these cells in CVID pathogenesis.

## Introduction

Common variable immunodeficiency (CVID) is an umbrella name for the most common symptomatic, but also the most heterogeneous, primary antibody deficiency in adults. Typical clinical features of this heterogeneous group of disorders include recurrent infections, decreased serum immunoglobulin (Ig) and impaired specific antibody (Ab) responses to vaccines reflecting impaired B cell responses (1). Diagnosis criteria recently defined by the European Society for ImmunoDeficiencies include at least one of the following: increased susceptibility to infections, autoimmune manifestations, granulomatous disease, unexplained polyclonal lymphoproliferation, or affected family member with antibody deficiency. Moreover, the following parameters should be present to confirm the diagnosis: diagnosis after the age of 4 years, no evidence of profound T-cell deficiency, deficit in serum Ig (multiple classes) not explained by other known causes, and impaired vaccination responses or low switched memory B cells (smB cells) (2, 3). CVID has a complex genetic basis, with monogenetic causative forms and genetic predispositions (4), as reviewed in Cunningham-Rundles (5). Some CVID forms are inherited, but family members of CVID patients are usually normal and not all individuals who inherit a gene mutation associated with CVID will develop the disease (6). Nevertheless, a genetic cause has been identified in about 25% of CVID patients using next-generation sequencing. As examples, mutations in several genes encoding for B cell receptor complex associated proteins, B cell activating factor receptor (BAFF-R), inducible co-stimulator (ICOS), cytotoxic T-lymphocyte-associated protein 4 (CTLA-4), phosphatidylinositol 3-kinase (PI3K), and in lipopolysaccharide-responsive beige-like anchor (LRBA) protein or more recently the NFκB family have been described (5–7). Mutations in the *TNFRSF13B* gene encoding the transmembrane activator and CAML interactor (TACI) are found in 8–10% of patients (8) but relatives to CVID patients with mutations in TACI display normal levels of Ig. The identification of mutations in genes encoding factors important in B cell generation or differentiation is not surprising, as CVID patients present abnormalities in the B cell compartment. In fact, impaired B cell differentiation is a hallmark of the disease and, despite normal levels of total B cells in most cases, post-germinal center (GC) B cells are defective and patients harbor lower levels or absence of smB cells (9, 10). Consequently, multiple CVID classifications based on B-cell phenotype have been proposed. On top of these classifications, two groups of patients are often described in the literature, namely one comprising patients that show only recurrent infections, and the other with patients harboring at least one of the following complications: (i) benign, granulomatous, or malignant lymphoproliferation, (ii) chronic enteropathy, and (iii) autoimmune manifestations. Moreover, a report in 2014 of the largest cohort of CVID patients studied so far highlighted that an early-onset of CVID (before the age of 10) is associated with infections (especially pneumonia) rather than other complications, suggesting two distinct disease entities (11). The pathogenesis leading to immune disorders of CVID is still poorly understood, but functional impairments in multiple immune cell types may be responsible for some of the pathophysiology of CVID.

## Immunological Features of Cvid Patients With Non-Infectious Complications

More than half of the patients harbor non-infectious complications causing increased morbidity and mortality (12). Cancers occur in 20% of CVID patients, the majority of cancers being lymphoma (13, 14). More than 25% of CVID patients have autoimmune complications (15). Immune thrombocytopenia (ITP) and autoimmune hemolytic anemia are the most frequent disorders, but many others such as vitiligo, pernicious anemia, systemic lupus erythemateous, rheumatoid arthritis, antiphospholipid syndrome, juvenile idiopathic arthritis, Sjögren's disease, psoriasis, thyroiditis, uveitis, and vasculitis can also be found in CVID patients (15). As impairment of B cell maturation is a hallmark of the disease, these cells have drawn a lot of attention. Wehr et al. have shown a significant decrease in isotype-switch memory B cells in patients with non-infectious complications such as autoimmunity, granulomatous disease, lymphoid hyperplasia, or splenomegaly (12). Intriguingly, despite defects in B cell differentiation and serum Ig, CVID patients develop autoantibodies and autoimmune manifestations. Such a paradigm might be due to a default in specific checkpoints for autoreactive B cells, although this hypothesis has yet to be proven. Interestingly, autoimmunity in CVID has been associated with the presence of CD21^low^ B cells, an “innate-like” population expressing low levels of CD38 but exhibiting autoreactivity (16, 17). Moreover, an increase of CD21^low^ B cells has been observed in CVID patients presenting immune thrombocytopenia (ITP) (18). It has been shown that CD21^low^ cells may develop from memory B cells under chronic inflammatory conditions and are present at high levels in autoimmune patients (19). These observations suggest a role for these CD21^low^ smB cells in the development of autoimmune complications observed in CVID patients, but this possibility remains to be explored.

Beyond the impairment of B cell functions, numerous immune alterations have been described in CVID patients with non-infectious manifestations. For instance, dysfunctions in monocytes/macrophages, dendritic cells (20), NK cells and innate lymphoid cells (ILCs) have been reported. Monocytes have impaired antigen-presenting capacities but increased capacity to produce reactive oxygen species or IL-12 (21). By contrast, IL-12 production by dendritic cells from CVID patients is lower than that of healthy donors, reflecting a defective maturation of these cells (22, 23). Two studies have reported a decrease in ILCs, either in CD127^+^CD90^+^ ILCs (24) or in ILC2s (25). By contrast, a study from Cols et al. (26) shows an expanded population of ILCs harboring an IFNγ signature in patients with non-infectious complications, suggesting that ILCs may be a critical source of IFNγ in these patients. Overall, defining the roles of ILCs in CVID pathogenesis still needs further investigation.

Numerous studies have reported abnormalities in the T-cell compartment [as reviewed in (27)], which is not surprising given the central role of T cells, especially CD4 T cells, in B cell activation and differentiation into memory and Ig-producing cells. Patients with complications usually have low numbers of naive CD4 T cells but increased activated CD4 T cell counts (28–30), defective T cell functions (lower proliferative capacities, abnormalities in cytokine production) and reduced levels of regulatory T cells (31). Given their function as B helper cells, Tfh represent a CD4 T cell subset of great interest in CVID pathogenesis and will now be discussed.

## Overview of Tfh Cell Functions

Tfh are a CD4 T cell subset specialized in providing B cell help. They are essential for B cell differentiation into Ig-producing plasma cells and for generation of memory B cells. Tfh are characterized by a unique set of molecules associated with their functions. The hallmark of Tfh is CXCR5 expression, which allows their migration into GC follicles of secondary lymphoid organs through the attractive effect of the CXCL13 chemokine (32–34). Moreover, they express the transcription factor B cell lymphoma 6 (BCL-6), the co-receptors CD40L, programmed cell death 1 (PD-1) and ICOS, and they produce IL-21 (34), all of which being involved in their functions.

Mouse models have led to a better understanding of Tfh biology over the past decade and these discoveries have already been reviewed (34–37). Here, we will focus on human Tfh and their subsets. In fact, recent studies have considerably increased our knowledge of the human counterpart. The discovery of human circulating Tfh within the memory CD4 T cell compartment has enabled a better understanding of these cells, since access to blood samples is much easier than access to secondary lymphoid organs such as spleen from cadaveric organ donors or tonsils from children (38). They are considered as memory cells and reflect the *bona fide* Tfh present in GC counterparts, even if they lack BCL-6 and ICOS expression. Interestingly, a recent and elegant study from Vella et al. comparing Tfh from LN, thoracic duct lymph and blood shows that these cells share TCR clonotype, phenotype and transcriptional signatures, thus reinforcing the idea that the examination of circulating cells reflects what happens in GC (39). Based on the expression of the chemokine receptors CXCR3 and CCR6, Morita et al. have identified three subsets of Tfh harboring different functions and affiliated with the classical helper subsets Th1, Th2, and Th17 (38) (Table 1). Tfh 1 are CXCR3^+^CCR6^−^, express T-bet and produce IFNγ; Tfh 2 are CXCR3^−^CCR6^−^, express GATA3 and produce IL-21 and IL-4; and Tfh 17 are CXCR3^−^CCR6^+^, express RORγT and produce IL-21 and IL-17A. More importantly, these subsets are divided into two groups based on their B helper cell functions, in particular their capacity to induce naive B cells to produce Ig: Tfh 2 and Tfh 17 are considered efficient helper cells, while Tfh 1 are non-efficient helpers (38, 42, 43). Based on CCR7, PD-1 and ICOS expression, these subsets can be further divided into different functional subpopulations, leading to the proposition by Ueno's group to include all these markers for human blood phenotyping of Tfh (44, 45). ICOS^+^PD-1^high^CCR7^low^ Tfh are activated and could be considered as effectors. For instance, following influenza vaccination, Tfh 1 (known as non-helpers) can be activated to express ICOS and high levels of PD-1, also correlating with antibody responses. This means that they are able to help memory B cells *in vitro*, showing then a limited B helper cell function (46). Similarly, CXCR3^+^ Tfh expressing high levels of PD-1 correlate with neutralizing antibody responses in HCV patients (47). In contrast, Martin-Gayo et al. reported that neutralizing antibodies in HIV controllers correlate with the presence of CXCR3^+^PD1^low^ Tfh, but that these cells might be precursors of PD-1^high^ cells (48). Another subset of Tfh, the T follicular regulatory cells (Tfr cells) comprising a population of natural regulatory T cells that express FoxP3, BCL-6, and CXCR5, has been identified in mice. This subset seems important for the regulation of the GC reaction by limiting the number of Tfh and B cells in GC or terminating the GC response (49–51). The biology of human Tfr cells is not well-known. In human tonsils, the number of FoxP3^+^ Tfr in GC is lower than it is in mice (35). Circulating FoxP3^+^ Tfr have been described (40). Cañete et al. identified a population of IL-10 producing human Tfh expressing CD25 but lacking FoxP3 in tonsils and capable of dampening IgE responses, thereby suggesting a possible role for these cells in atopic diseases (41). Altogether, despite several studies focused on Tfh biology over the past decade, the functions of each human subset are not fully discovered yet.

**Table 1:** Main characteristics of circulating Tfh subsets.

	**Tfh 1**	**Tfh 2**	**Tfh 17**	**Tfr**
B helper function	±	+	+	
Surface marker	CXCR3	–	CCR6	CD25^high^ CD127^low^
Transcription factor	T-bet	Gata3	RORγT	FoxP3^±^^*^
Cytokine profile	IL21^low^ IFNγ	IL21; IL4; IL13	IL21; IL17; IL-22	IL-10

*The main characteristics of the circulating CD4^+^CD45Ra^−^CXCR5^+^ follicular helper T cell subsets are described*.

* *Both FoxP3+ and FoxP3 Tfr have been reported (40, 41)*.

Mouse Tfh differentiation is a multi-step process involving several signals, with a priming by dendritic cells (DC), or eventually B cells (52), in the T cell zone of secondary lymphoid organs, followed by migration of the pre-Tfh to the T-B border and maturation into *bona fide* GC Tfh requiring B cells (53). Human Tfh differentiation has yet to become fully understood. IL-12 (54, 55), TGFβ (56), Activin A (57), and OX40L (58, 59) are key regulators of this process. Dermal CD14^+^ DCs have been found as the best skin DC subset to drive Tfh differentiation (60). Others have identified CD1a^+^ dermal DCs and Langerhans cells as able to polarize CD4 T cell into IL-21 producer cells (61, 62). Recently, Durand et al. have uncovered tonsil cDC2 as the best Tfh polarization inducer among the DC subsets they tested, and have shown that the interaction with tonsil macrophages located in B cell follicles is necessary for optimal Tfh function (63).

Tfh are involved in numerous biological processes of health and disease, as reviewed in Ueno et al. (35), Crotty (36), and Ma and Deenick (64). They are involved in protection against numerous pathogens through the induction of Ab responses and vaccine-induced immunity, as well as in autoimmune diseases or HIV infection. The role of Tfh in human primary immunodeficiency has already been well documented and reviewed (64, 65). For instance, distinct monogenic mutations in *STAT3, CD40LG, BTK, IL10R*, or *NEMO* that lead to different types of primary immune deficiency are associated with decreased circulating Tfh number (66).

## Tfh and Cvid

As mentioned earlier, CVID is defined by B cell defects leading to low levels of serum Ig and impaired Ab responses. Nevertheless, defects in other immune cells are also present. Given their role as B helper cells, it is of interest to analyze Tfh subsets in CVID patients. One series of evidence for Tfh involvement in CVID pathogenesis is given by genetic analysis. The most striking is the rare deficiency in inducible T-cell COStimulator (ICOS), a co-receptor expressed by T cells. In these patients, B cells are genetically normal but do not receive optimal help from T cells, which leads to impaired T-cell dependent B-cell activation, absence of memory B cells, and failure in class-switching leading to hypogammaglobulinemia (67–69). Warnatz et al. studied nine patients with ICOS deletion and showed that combining all clinical features of the patients outlines the full range of associated complications to CVID (69). Interestingly, Bossaller et al. showed that ICOS deficiency is associated with a defect of Tfh in germinal centers (68), showing that ICOS is essential for Tfh generation in humans as well as in mice (70). Similarly, patients with a mutated *NFKB2* gene showed decreased levels of circulating Tfh (71, 72). By contrast, Romberg et al. showed that a single *TACI* mutation leads to increased levels of circulating Tfh in CVID patients which correlate with levels of anti-nuclear antibodies suggesting that Tfh may favor autoreactive B cell activation (73). Interestingly, Ellyard et al. also observed increased Tfh, particularly circulating Tfh 1, in *TACI* mutant patients and of PD-1^hi^ CCR7^lo^ Tfh cells in CTLA4 mutant patients (74).

Interestingly, our group (75) and others (76–78) observed an increase of circulating Tfh (memory CXCR5^+^ CD4 T cells) in CVID patients harboring non-infectious complications. Moreover, Tfh expressing PD-1 were present at higher levels in CVID patients with complications (75–78). Patients classified as smB^−^ based on the EUROClass have <2% of switched memory B cells among circulating CD19^+^ cells (12). Interestingly, smB^−^ patients have higher levels of circulating Tfh (77) [which is even more pronounced in the smB^−^ CD21^low^ subgroup (78)] than smB^+^ patients. The switched memory B cell population (IgG^+^) contains some autoreactive B cells in normal adults (79), and CD21^low^ memory B cells are increased in several autoimmune contexts (18). One can then hypothesize that smB cells in CVID patients, despite their low levels, contribute to autoimmunity, so Tfh could participate to autoimmune manifestations through their role as smB cell inducers. Nevertheless, patients with autoimmune complications present similar levels of Tfh or Tfh subtypes to patients harboring other types of comorbidities (75), meaning that further experiments are needed to determine the impact of Tfh on autoreactive Ab generation in CVID patients.

As explained earlier, Tfh can be divided into two subsets: the non-efficient helper Tfh 1 and the efficient helpers Tfh 2 and Tfh 17. Interestingly, we (75) and others (77, 78, 80) highlight a specific increase of the circulating Tfh 1 only in non-infectious CVID patients. Moreover, CXCR3^+^ (75) or T-bet^+^ (78) cells were amplified in secondary lymphoid organs of CVID patients, suggesting that the blood observations reflect the GC counterpart. In contrast, Th17-oriented Tfh were decreased. An increase in CD25^+^CD127^−^CXCR5^+^PD-1^+^ cells was observed, but these cells do not present regulatory functions and still need to be further characterized (80). Tfh 1 are not efficient B helper cells, partly due to their poor production of IL-21 (38). The combination of IL-21 and CD40 stimulation is able to restore Ig production and to improve memory B cell survival in *in vitro* settings using cells from CVID patients (81, 82). Moreover, addition of IL-4 and IL-21 (cytokines produced by Tfh 2) improved IgG production in some patients (83). Thus, the imbalance between Tfh subsets, stable over time (75), could lead to poor IgG production. As Tfh 1 are good IFNγ producers and are increased in patients, one may hypothesize involvement of this cytokine in CVID pathogenesis. Surprisingly, even though two groups observed enhanced IFNγ production by Tfh in CVID patients (77, 78), Le Coz et al. did not, rather finding increased IL-21^+^ cells and accordingly efficient helper B cell function in CVID Tfh despite observing a Tfh 1/Tfh 2-17 imbalance (80). Moreover, studies on putative IFNγ function in CVID are also puzzling. In fact, Desjardin et al. reported that addition of IFNγ to cultured B cells from CVID patients did not modulate IgG production (83), while Unger et al. showed that exogenous IFNγ reduced IgG and IgA production in T/B co-cultures (78). Moreover, the impact of IFNγ on CD21^low^ cell generation and/or on autoreactive B cell activation has not been directly addressed, therefore still awaiting determination. Altogether, these data highlight that more experiments are necessary to determine Tfh 1 functions and putative IFNγ implication in the diverse clinical manifestations of CVID.

A question one may ask is the origin of the skewed Tfh populations in CVID patients. A recent study from Le Coz et al. highlighted that part of the naïve CD4 T cells from CVID patients with autoimmune cytopenias (AIC) are skewed toward a follicular commitment based on their expression of specific markers (CXCR5, PD-1, CCR7, CD38, ICOS, T-cell factor 1). In addition, some recently identified thymic emigrant cells (defined as CD45RA^+^CD31^+^) express CXCR5 and PD-1 in CVID patients with AIC (80). These data suggest that CD4 T cells present follicular aspects as early as thymic egress stage. Moreover, Tfh can differentiate from naive CD4^+^ T cells by interacting with different dendritic cell subsets or under the influence of several cytokines such as IL-12 (55), TGFβ (56) or Activin A (57). Notably, Martinez-Pomar et al. reported high amounts of IL-12 in the sera of CVID patients (84), which was not confirmed by Le Coz et al. (80). By contrast, they found an increase in plasma levels of Activin A, correlating with circulating Tfh frequencies. They also observed increased ICOSL expression on monocytes and demonstrated that endotoxemia is involved in Tfh differentiation in CVID patients with AIC (80). Altogether, despite recent studies, the mechanisms leading to the imbalance of Tfh 1 vs. Tfh 2/Tfh 17 in CVID patients still need to be fully decoded.

## Conclusion

Evidence from the literature strongly suggests a role for Tfh in pathogenesis of the more severe forms of CVID, but more experiments are necessary to determine the mechanisms involved. A better understanding of these mechanisms would be of great interest to apprehend the immune context in CVID patients harboring non-infectious complications.

## Author Contributions

CL and DD wrote and edited the manuscript. SL, PB, and J-FV contributed to writing and critically revised the paper. All authors read, corrected, and approved the final manuscript.

### Conflict of Interest

The authors declare that the research was conducted in the absence of any commercial or financial relationships that could be construed as a potential conflict of interest.
